# Emerging Roles for the RNA-Binding Protein HuD (ELAVL4) in Nervous System Diseases

**DOI:** 10.3390/ijms232314606

**Published:** 2022-11-23

**Authors:** Beatrice Silvestri, Michela Mochi, Maria Giovanna Garone, Alessandro Rosa

**Affiliations:** 1 Department of Biology and Biotechnologies “Charles Darwin”, Sapienza University of Rome, 00185 Rome, Italy; 2 Center for Life Nano & Neuro-Science, Fondazione Istituto Italiano di Tecnologia (IIT), 00161 Rome, Italy; 3 Department of Stem Cell Biology, Murdoch Children’s Research Institute, The Royal Children’s Hospital, Melbourne, VIC 3052, Australia

**Keywords:** HuD, amyotrophic lateral sclerosis, Alzheimer’s disease, Parkinson’s disease, RNA-binding protein, ELAVL4

## Abstract

The main goal of this review is to provide an updated overview of the involvement of the RNA-binding protein (RBP) HuD, encoded by the ELAVL4 gene, in nervous system development, maintenance, and function, and its emerging role in nervous system diseases. A particular focus is on recent studies reporting altered HuD levels, or activity, in disease models and patients. Substantial evidence suggests HuD involvement in Parkinson’s disease (PD), Alzheimer’s disease (AD), and amyotrophic lateral sclerosis (ALS). Interestingly, while possible disease-causing mutations in the ELAVL4 gene remain elusive, a common theme in these diseases seems to be the altered regulation of HuD at multiple steps, including post-transcriptional and post-translational levels. In turn, the changed activity of HuD can have profound implications for its target transcripts, which are overly stabilized in case of HuD gain of function (as proposed in PD and ALS) or reduced in case of decreased HuD binding (as suggested by some studies in AD). Moreover, the recent discovery that HuD is a component of pathological cytoplasmic inclusion in both familial and sporadic ALS patients might help uncover the common molecular mechanisms underlying such complex diseases. We believe that deepening our understanding of the involvement of HuD in neurodegeneration could help developing new diagnostic and therapeutic tools.

## 1. HuD Functions in Neuronal Development, Synaptic Plasticity and Regeneration

The RNA-binding protein (RBP) HuD, encoded by the *ELAVL4* gene, is a member of the Hu protein family, which includes homologs of the Drosophila elav (embryonic lethal abnormal vision) gene, while HuB (ELAVL2) is expressed in neurons and gonads, and HuR (ELAVL1) is ubiquitously expressed in all tissues, HuC (ELAVL3) and HuD are exclusively expressed in neurons [1]. HuD is considered one of the most important regulatory factors in the nervous system, governing many neuronal processes such as development, plasticity, and functionality. In this context, HuD exerts its regulatory functions through tightly controlling mRNA metabolism, including neuronal mRNA stability, localization, degradation, and translation. HuD mRNA is detectable since the initial stages of brain development, suggesting an early role in the regulation of nervous system formation [2]. Indeed, one of its leading roles is in the context of neuronal commitment by suppressing neuroblast proliferation [3]. *HuD* knockout mice showed a significant increase in the self-renewal and loss of differentiation of neural precursors [4]. Moreover, *HuD*-deficient mice showed higher levels of apoptosis and an increased number of subventricular proliferative zones, suggesting that finely regulated *HuD* expression is essential for promoting differentiation [4]. HuD downregulation resulted in a remarkable inhibition of neurites outgrowth [5,6]. Contrarily, up-regulated HuD levels in embryonic stem cells produced a significant increase in the rate and length of growing neurites, and in the number of cells with long extensions [6,7]. This evidence supports the key role of HuD in neurite morphological development, specifically in axonal and dendritic elongation [6,8,9]. The HuD protein is predominantly localized in the cytoplasm, where it acts at the post-transcriptional level by stabilizing its target mRNAs. In neurons, important HuD targets such as *GAP-43*, *Tau*, and *NEURITIN1* (*NRN1*) are involved in growth cone development and cytoskeletal assembly [5,6,8,9,10,11]. In particular, GAP-43 is crucial for axonal growth during nervous system development [6,8,12]. HuD promotes neurite outgrowth by increasing *GAP-43* mRNA levels by binding its 3’ untranslated region (3’UTR) [6]. Accordingly, a decrease in HuD levels led to an acceleration of the *GAP-43* mRNA degradation rate and resulted in defective neurite outgrowth during differentiation [8]. The AU-rich element (ARE) present in the 3’UTR is sufficient for *GAP-43* mRNA localization and translation in the axon region through the interaction with a complex formed by HuD and ZBP1 [13]. HuD also plays a role in supporting axonal recovery after neurites damage by stabilizing key target transcripts. Both GAP-43 and HuD levels are upregulated during post-injury axonal regeneration [13]. Another well-known HuD target is NRN1, which promotes axon extension during development and upon injury [9,14]. In addition, NRN1 expression has been detected in the earliest stages of ventral spinal cord development, suggesting its implication in the refining process of exuberant branches for establishing neuromuscular junctions (NMJs) [15]. Together these data suggest an indirect role for HuD during the development of neuromuscular synaptic connections.

## 2. HuD Structure and Functioning Mechanisms

**Figure 1:**
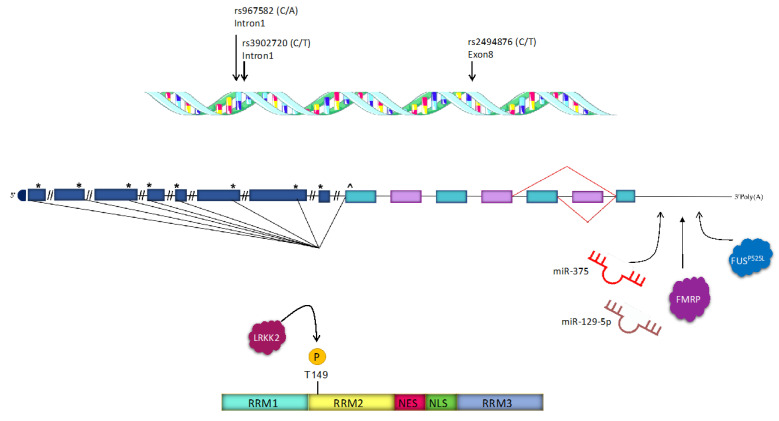
Schematic representation of HuD gene, mRNA, and protein. Top, a schematic representation of the *ELAVL4* gene in which the PD-linked SNPs are indicated. Middle, a schematic representation of *HuD* mRNA isoforms, showing coding exons (green and purple rectangles), alternative non-coding exon 1 (blue rectangles), and the favored (^) versus putative alternate translation start site (*). MicroRNAs and RBPs interacting with the 3’UTR are also displayed. Bottom, schematic diagram of HuD protein domains and the post-translational modification involved in PD. This figure was drawn using the vector image bank of Servier Medical Art (https://smart.servier.com/).

The *ELAVL4* gene, located on chromosome 1 in humans, is well conserved in vertebrates [3]. It spans ~146 kb of DNA and is divided into seven coding exons (E2 to E8) [1]. The complexity of the 5’ sequence of HuD transcripts, which encode different HuD N-termini, and the alternative splicing of exons 6 and 7 lead to the generation of several HuD mRNA isoforms [16] (Figure 1). The encoded protein is ~40–42 kDa in size and contains three RNA recognition motifs (RRMs). The linker region, housing the nuclear export (NES) and the putative nuclear localization signal (NLS), separates the second and the third RRM and contains multiple residues that are post-translationally modified [1]. RRM1 and RRM2 associate with target mRNAs by binding to AREs, which are commonly found in the 3’UTR of short-lived transcripts encoding proteins involved in cellular proliferation, differentiation, transcription, RNA metabolism, inflammation, and stress response [1,17]. The third RRM is also involved in ARE binding, but it interacts as well with long poly(A) tails of transcripts and with other proteins, mediating homo- and hetero-multimerization of Hu family members [1,16]. 

HuD variants differ in amino acid sequences of NLS or NES in the linker region, which play a crucial role in the temporal and spatial regulation of neuronal differentiation [16]. Indeed, the analysis of the sequence between RRM2 and RRM3 led to the identification of three variants, which are expressed in specific stages of neuronal differentiation and show variable localization patterns in cells, thus playing different roles according to their cellular compartment [18].

**Figure 2:**
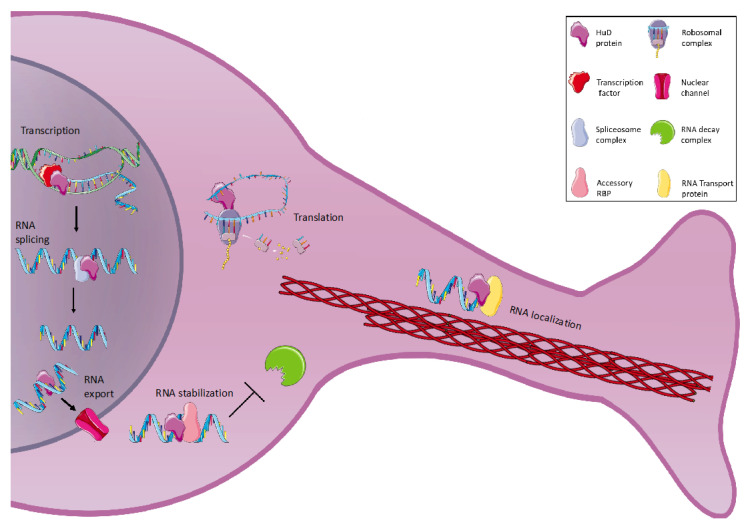
Schematic representation of HuD roles in neurons. In the nucleus, HuD cooperates with transcription factors for RNA synthesis, it is involved in RNA splicing through interaction with spliceosome complex, and it promotes mRNA export to the cytoplasm through nuclear pores. In the cytoplasm, HuD is involved in mRNAs translation and, in cooperation with other RBPs, it prevents mRNAs degradation and controls specific mRNA localization in neuronal compartments. This figure was drawn using the vector image bank of Servier Medical Art (https://smart.servier.com/).

HuD controls neuronal gene expression at multiple levels, including mRNA turnover, translation, splicing and localization [16] (Figure 2). Several studies demonstrated the role of HuD as a stabilizer of neuronal mRNAs, decreasing the deadenylation rate of its targets [3,17]. Since the deadenylation is the first step of mRNA degradation, followed by rapid and processive decay of the body of mRNA, HuD plays an important role in increasing mRNA half-life. Along with promoting mRNA stability, evidence supports the role of HuD in regulating both the localization and translation of its target transcripts [1]. As previously mentioned, a nuclear export signal in the linker region allows HuD to shuttle transcripts into the cytoplasm; in addition, the first two RRM domains and the linker region interact with the mRNA export receptor TAP/NXF1 [19]. 

With a few exceptions, HuD generally promotes the expression of target genes, enhancing their mRNAs’ translation (Table 1). HuD enhances cap-dependent translation by binding to eIF4A and the poly(A) tail of transcripts via its linker region and the third RRM domain. However, HuD can also repress some targets’ protein synthesis [1,16]. Moreover, HuD has been shown to control alternative splicing and alternative polyadenylation of neuronal transcripts. Several studies demonstrated that HuD, along with other neural ELAV proteins, can promote or suppress exon inclusion by interacting with (or antagonizing) splicing, transcription, and chromatin components. In addition, by blocking the association of the essential components of the cleavage and polyadenylation machinery, HuD can regulate alternative polyadenylation of target mRNAs, such as calcitonin/calcitonin gene-related peptide (CGRP). In this case, HuD binding is crucial to promote the neuron-specific CGRP pathway [20]. Finally, it has been recently reported that HuD can bind neuronal circular RNAs (circRNAs), thus participating in the regulation of networks comprising mRNAs, circRNAs, and microRNAs [21,22,23].

## 3. Possible Roles of HuD in Nervous System Diseases

**Figure 3:**
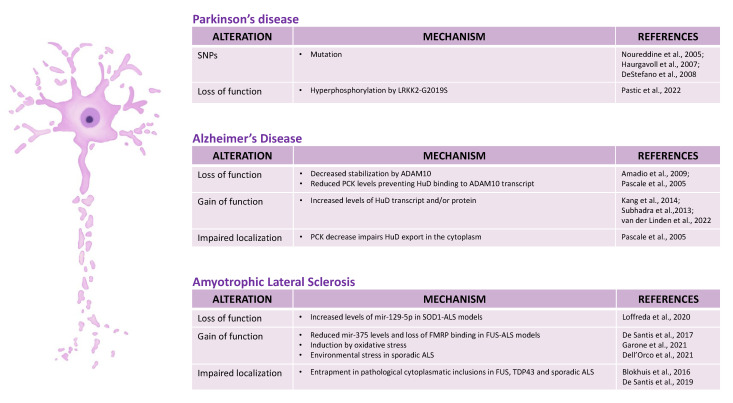
Summative figure of the evidence on the possible implication of HuD in major neurodegenerative diseases [25,26,28,44,46,47,48,49,50,51,52,53,54,55,56].

HuD functions have been extensively studied in neuronal development, plasticity, and regeneration. Recent studies, however, suggest that HuD misregulation might underlie neurological disorders, including neurodegenerative diseases such as Parkinson’s disease, Alzheimer’s disease, and amyotrophic lateral sclerosis (Figure 3). 

### 3.1. Parkinson’s Disease (PD)

PD is a neurodegenerative disease characterized by the loss of dopaminergic neurons caused by the aberrant accumulation of α-synuclein in the midbrain. The pathological etiology seems to be related to both genetic and environmental risk factors [57]. Interestingly, three different studies analyzed the genetic elements influencing the age-at-onset (AAO) of PD and among various candidates they reported HuD as a plausibly associated factor [46,47,48]. PARK10 is a genetic locus with a robust linkage signal associated with AAO of PD mapping on chromosome 1p, which hosts the ELAVL4 gene. Among nine different single-nucleotide polymorphisms (SNPs) found in the Caucasian population, two have been associated with the AAO of PD. Specifically, rs967582 is located in the first intron and rs2494876 is a non-synonymous SNP mapping in the coding region of exon 8 [46]. Another study examined the correlation between ELAVL4 SNPs and AAO of PD in Norwegian, United States (US) and Irish populations [47]. While no association was found in the first two populations, a strong link has been identified in the Irish one for two markers: the already mentioned rs967582 and another intronic SNP, rs3902720. Given the correlation between the Iceland population with late-onset PD and their Celtic origins, a possible Irish founder effect in the ELAVL4 association to PD has been hypothesized. A third study confirmed the strong correlation between PD and the rs967582 SNP, extending its relevance also in US and Norwegian populations [48]. Interestingly, the rs2494876 SNP consists of a serine substitution with a proline in the linker region between RRM2 and RRM3, with possible consequences in the protein function [17]. 

Increased activity of the Leucine-Rich-Repeat Kinase-2 (LRKK2), due to mutations such as the G2019S, is a major cause of familial PD. Pastic et al. recently demonstrated that LRKK2 can phosphorylate conserved serine and threonine residues in the RRM2 of neuronal ELAVLs, including HuD [28]. They proposed that hyperphosphorylation of the RRM2 by LRKK2-G2019S can alter the activity of HuD in PD. Specifically, phosphorylation of HuD RRM2 increases its binding to mRNA target. Notably, HuD hyperphosphorylation by LRRK2 has an inhibitory effect on its mRNA stabilizing activity, while it enhances its effects on mRNA splicing. Levels of several HuD targets, including genes involved in the mitochondrial organization, inflammation, and organelle trafficking, were accordingly altered in PD patients. 

### 3.2. Alzheimer’s Disease (AD)

HuD involvement in processes of learning and memory formation paved the way for studies on the impairment of its functions in AD, a neurodegenerative disease characterized by progressive loss of memory and cognitive functions. Amadio et al. showed a remarkable decrease in HuD levels correlated to an increase of β-amyloid (Aβ) aggregates in postmortem hippocampal tissues of AD patients [25]. They reported higher levels of pathogenic Aβ peptide with a length of 42 residues (Aβ1-42) compared with a shorter physiological form (Aβ1-40). This might be partly due to impaired *ADAM10* mRNA stabilization due to reduced HuD levels. *ADAM10* encodes for one of the most relevant α-secretases involved in APP cleavage for producing soluble and non-pathogenic AβPP. The presence of an ARE region in the *ADAM10* 3’UTR suggested possible post-transcriptional regulation by HuD [25]. Immunoprecipitation analysis demonstrated that *ADAM10* mRNA is indeed a HuD target [25]. In addition, HuD binding to the *ADAM10* ARE could be promoted by protein kinase C (PKC). PKC, which is decreased in postmortem AD brain tissues, is directly implicated in the activation of the α-secretase ADAM10, leading to an increase of the soluble AβPP production at the expense of Aβ fragments. Moreover, PKC promotes HuD export from the nucleus and its cytoplasmic and cytoskeletal localization, with important consequences in the up-regulation of HuD targets [49]. 

In contrast with the earlier study by Amadio et al. [25], who found decreased HuD levels in the hippocampus, other authors have later reported increased HuD levels in AD patients’ post-mortem samples. In particular, increased HuD levels have been detected in superior temporal gyrus by Kang et al. [26] and in the frontal cortex, possibly due to increased activation of the thyroid hormone pathway, by Subhadra et al. [50]. Regarding the underlying pathological mechanisms, Kang et al. investigated the association between HuD, the β-secretase BACE1, and the long noncoding RNA (lncRNA) *BACE1AS* [26]. β-secretase enzymes are crucial for Aβ production and represent possible druggable targets for AD treatment. *BACE1AS* acts as a *BACE1* expression enhancer thanks to sequence complementarity to *BACE1* mRNA. HuD binding to *BACE1AS* increases the levels of this lncRNA, thus indirectly promoting *BACE1* stabilization and translation [26]. Accordingly, increased levels of HuD were mirrored by increased *BACE1AS* and *BACE1* mRNA levels in AD brains. Moreover, HuD overexpressing mice showed increased amounts of Aβ, APP, BACE1, and *BACE1AS* in the hippocampus, cortex, and cerebellum [26]. Collectively, these data further support the implication of HuD in APP cleavage control during AD progression. 

Transcript levels of another HuD target, neuroserpin, are increased in AD brains [50]. Neuroserpin is an inhibitor of tissue plasminogen activator (tPA). tPA inhibition leads to a dramatic reduction of plasmin protease activity, which regulates and degrades β-amyloid plaques for safeguarding brain homeostasis. Thus, in AD patients’ brain, increased HuD activity might lead to an aberrant rise in neuroserpin protein levels [50]. 

A recent study analyzed the effects of HuD loss- and gain-of-function in an iPSC-based AD model [51]. HuD overexpression rescued the AD-associated phenotype, up-regulating some specific APP splicing isoforms and reducing the Aβ1-42/Aβ1-40 ratio. In particular, the authors showed a significant increase in the APP695 splicing isoform, which is downregulated in AD, at the expense of the APP751 and APP770 isoforms. In addition, HuD overexpressing cortical neurons showed Aβ1-42 reduced levels compared with the β-amyloid protective Aβ1-40 counterpart. Transcriptional and proteomic analysis in overexpressing neurons revealed that HuD regulates several cellular pathways. Particularly noteworthy is “axogenesis signaling”, including axonal guidance and synaptogenesis regulation. Moreover, aberrant DNA damage, cell cycle re-entry and mitochondrial pathways were impaired in HuD knock-down and AD conditions.

### 3.3. Amyotrophic Lateral Sclerosis (ALS)

ALS is a complex neurodegenerative disease primarily caused by motoneurons’ (MNs) progressive loss, with considerable genetic and phenotypic heterogeneity. Indeed, clinical and basic research evidence suggests multiple contributing factors, with important but varied genetic components and a complex onset. Recent evidence suggests that altered HuD activity might be a common underlying pathomechanism in both familial (fALS) and sporadic (sALS) ALS. 

In 2011, two independent laboratories studied HuD in the context of the MN. The authors showed how the interaction between HuD and survival of motor neuron (SMN) could rescue motor neuron defects in spinal muscular atrophy, albeit with different mechanisms [9,58]. HuD was then found among the interactors of ALS-linked factors, including the RBPs Fused in sarcoma (FUS) and TAR DNA binding protein 43 (TDP-43), in neuroblastoma cells [55]. More direct evidence of the possible involvement of HuD in ALS was shown in 2017, when our laboratory performed a transcriptome profiling in induced pluripotent stem cells (iPSCs)-derived MNs. HuD transcript and protein levels were significantly increased in MNs carrying a severe ALS-linked mutation in the FUS gene (P525L) [53]. This may be partly due to the fact that miR-375, a microRNA negatively regulating HuD expression, was downregulated in FUS mutant MNs. Notably, miR-375 involvement in ALS might extend to sporadic ALS, since its levels were altered in a sALS mouse model [59]. Other microRNAs might be involved in HuD regulation in ALS. For instance, a mouse model carrying an ALS mutation in the Cu/Zn SuperOxide Dismutase 1 (*SOD1*) gene showed increased levels of miR-129-5p and decreased HuD levels [52]. 

Subsequent studies suggested that, beyond microRNAs, the molecular mechanisms underlying HuD altered levels in ALS might involve multiple levels of regulation. By taking advantage of photoactivatable ribonucleoside-enhanced crosslinking and immunoprecipitation (PAR-CLIP), we found that ALS mutant FUS, but not the wild-type protein, binds the *HuD* mRNA 3’UTR [56]. Mutant FUS binding to 3’UTRs generally correlates with increased protein levels of its targets [60]. In the case of HuD, the molecular mechanism involves the RBP FMRP (fragile X mental retardation protein). In MNs, FMRP directly binds the 3’UTR of HuD and acts as a negative regulator of HuD translation [54]. Mutant FUS binding to the same sites intrudes on this function, leading to increased HuD translation [54]. In turn, HuD targets NRN1 and GAP-43 were increased in FUS-ALS human and mouse models [54,61]. Accordingly, increased axon branching, arborization, and faster growth upon injury were observed in FUS mutant cells. These axonal phenotypes were rescued by dampening NRN1 levels in FUS-P525L MNs, demonstrating that this phenotype is a consequence of increased NRN1 [54]. New evidence suggests that the effects of HuD upregulation on its target genes in ALS might extend beyond NRN1 and GAP-43. By taking advantage of human iPSC-derived MNs cultured in compartmented chambers and RNA profiling by digital colour-coded molecular barcoding in soma and neurites, it was found that HuD overexpression in FUS-WT MNs produces changes strikingly similar to those observed in mutant FUS MNs, especially for the expression of a set of genes involved in synaptic transmission and neuron development [62]. This finding supports the importance of HuD in the context of a complex regulatory RBP network, which is in place in normal MNs and disrupted upon FUS mutation (and possibly also in sALS, see below). Accordingly, Tebaldi et al. showed that genes associated with MN diseases, such as ALS and spinal muscular atrophy (SMA), were enriched among HuD targets and that HuD overexpression in NSC-34 cells (a mouse hybrid line between MNs and neuroblastoma) increases their translation [63]. Interestingly, it has been recently reported HuD can bind and regulate the expression of neuronal circRNAs [22]. Since several circRNAs were deregulated upon FUS loss of function or mutation in MNs [64], this evidence suggests the possibility that the activities of these two RBPs might converge also on this class of transcripts, with implications for ALS.

HuD might play a role also at late disease stages as it was detected as a component of pathological cytoplasmic inclusions in FUS and TDP-43 ALS patients’ postmortem samples [55,56]. HuD capturing in these macromolecular aggregates, which represent a hallmark of the pathology, might occur via direct protein-protein interaction with mutant FUS (and possibly other protein components of the inclusions), through prior recruitment of HuD into stress granules (which are thought to represent precursors of the aggregate), and/or by binding to RNA molecules involved in aggregate formation [56,65]. Interestingly, the co-expression of mutant FUS and HuD in HeLa cells is sufficient to induce the formation of stiff cytoplasmic speckles, which might represent an early stage during aggregate formation [56].

HuD involvement in ALS might extend to the sporadic cases devoid of any mutation in known ALS-linked genes. We have detected HuD in pathological inclusions of sALS patients, colocalizing with a phosphorylated form of TDP-43 typically associated with the pathology [56]. The Cereda and Perrone-Bizzozero laboratories recently demonstrated that HuD binds the 3’UTR of *SOD1* mRNA increasing its levels during oxidative stress [44]. Notably, increased mRNA and protein levels of both SOD1 and HuD were reported in the motor cortex of sporadic ALS patients, possibly due to increased HuD binding to *SOD1* mRNA [44].

## 4. Conclusions and Future Perspectives

HuD involvement in neurological diseases was initially proposed when SNPs in the ELAVL4 gene were associated with PD. Since then, increasing evidence points to a possible role for this RBP in other neurodegenerative diseases as well. This is not unexpected, given its multiple functions in the nervous system. However, strong genetic evidence of HuD mutations associated with AD and ALS is still missing, and even for PD a causal effect of the aforementioned SNPs is still uncertain.

Recent studies suggest that in neurodegenerative diseases rather than being mutated per se, HuD functions could be altered due to impairment of the transcriptional, post-transcriptional and/or post-translational regulatory mechanisms that ensure, in normal neurons, proper levels of expression and activity. In PD, this would occur due to mutations in LRKK2, a kinase that phosphorylates HuD and other neural ELAVLs regulating their RNA binding ability. Altered HuD activity has been also proposed to play a role in AD. In this case, several studies have shown either decreased or increased HuD levels in post-mortem patients’ brain samples [25,26,50]. Such apparently conflicting evidence might result from the different brain regions analyzed (hippocampus or cortex). Moreover, it has been proposed that both a loss-of-function (in an iPSC-based model) and an aberrant gain-of-function (in a mouse) of HuD might trigger pathological mechanisms in AD [26,51]. Collectively, it is conceivable that an alteration of HuD levels in opposite directions might occur at different disease stages and/or in different brain regions in AD patients. In any case, both increased and decreased activity of HuD might produce a significant effect on multiple targets involved in AD pathogenesis.

In MNs, the 3’UTR of the HuD transcript represents an important regulatory element, to which direct binding has been shown for several microRNAs and RBPs. ALS-linked RBPs FUS and TDP-43 are involved in microRNA biogenesis. Therefore, impairment in the production of specific microRNAs might, at least in part, cause an aberrant increase of HuD protein levels in ALS patients’ MNs. Moreover, recent evidence that FMRP negatively regulates HuD translation in MNs suggests that, in normal conditions, keeping low levels of HuD is crucial for these cells. Thus, HuD aberrant upregulation due to environmental (e.g., oxidative stress) or genetic (FUS or TDP-43 mutation) triggers might represent an early pathogenic event with important consequences on the expression of several other genes involved in MN diseases. We and others have also reported that HuD is a component of pathological inclusions found in fALS (TDP-43 and FUS) and sALS. Notably, most of the proteins associated with these aggregates are ubiquitously expressed, while HuD is a neural-specific factor. Whether HuD represents a mere bystander or an active trigger in toxic aggregate formation is still unknown. Answering this question might lead to developing novel therapeutic strategies for ALS based on HuD targeting.

In conclusion, increasing evidence of alteration of HuD levels and/or activity in multiple neurodegenerative diseases lays the basis for the rational design of new diagnostic and therapeutic approaches. However, there are still important aspects that need to be clarified. A relevant point is related to the isoforms produced by alternative splicing, transcriptional start sites and cleavage/polyadenylation sites that are expressed in physiological and pathological conditions. Most of the studies have been focused so far on major HuD isoforms, but we cannot exclude that minor isoforms, possibly with different intracellular localization and/or RNA binding activity, play any role in disease. Notably, alternative cleavage and polyadenylation can produce transcripts with shorter 3’UTRs, which would escape from negative regulation by miRNAs (such as miR-375) and RBPs (such as FMRP). Similarly, post-translational modifications, such as phosphorylation, that might alter HuD activity have been rarely taken into consideration in previous studies. We believe that better characterization of HuD mRNA and protein species expressed at different disease stage, and in different brain regions, would provide important information to better clarify its contribution to disease. Another important advancement would be the characterization of HuD targetome in the specific cell type affected by each neurodegenerative disease, possibly at different disease stages. To this regard, human iPSCs, which can be derived from patients, modified by gene editing and differentiated into (virtually) any cell type, could represent a useful tool for RNA-immunoprecipitation or cross-linking and immunoprecipitation (CLIP)-based approaches. In parallel with these basic studies, we propose that strategies for modulating HuD levels (or activity) should be taken into account for developing new therapeutic approaches for neurodegenerative diseases. In this regard, an interesting opportunity is provided by recent pre-clinical and clinical studies based on the use of artificial oligonucleotides, such as aptamers or antisense oligonucleotides (ASOs) [66,67]. Aptamers are synthetic modified nucleic acids able to bind with high affinity a molecular target. An important advancement in the field comes from the possibility to design aptamer sequences which are highly specific for a given RBP, thus allowing to interfere with its activity and/or visualize it by microscopy with subcellular resolution [68]. ASOs can be used to modulate gene expression at the post-transcriptional level by different mechanisms, e.g., splicing modulation or RNase H-mediated knock-down. Importantly, this technology has been already translated into an effective drug for neurodegeneration [69].

## Figures and Tables

**Table 1:** **HuD target transcripts.** The table shows a list of known HuD target transcripts. In the “Function” column, + indicates upregulation and – indicates downregulation.

Target	Region	Sequence	Regulatory Mechanism	Function	Reference
AChE	3’UTR	AU-rich element	mRNA stability	+	[10]
AChE	3’UTR	AU-rich element	mRNA stability	+	[24]
ADAM10	3’UTR	AU-rich element	mRNA stability	+	[25]
APP	3’UTR		mRNA stability	+	[26]
APP	Intron	U-rich element	Alternative splicing	+	[27]
α-synuclein (SNCA)	3’UTR	U-rich element		+	[28]
BACE1	3’UTR	U-rich element	mRNA stability	+	[26]
BACE-AS	-	U-rich element		+	[26]
BDNF	3’UTR	AU-rich element	mRNA stability	+	[29]
CGPR	Intron	U-rich element	Alternative splicing	+	[20]
CaMKIIα	3’UTR	AU-rich element	mRNA stability	+	[30]
CDKN1A	3’UTR	U-rich element	mRNA stability	+	[31]
cirHomer1a	-	AU-rich element	Expression and transport	+	[22]
GAP-43	3’UTR	AU-rich element	mRNA stability	+	[32]
GAP-44	3’UTR	AU-rich element	Transport	+	[13]
Gls	Intron	GU-rich element	Alternative splicing	-	[33]
LRRK2	3’UTR	U-rich element		+	[28]
MYCN	3’UTR	AU-rich element	mRNA stability	+	[34]
MYCN	3’UTR	AU-rich element	mRNA stability	+	[35]
NEP	3’UTR	AU-rich element	mRNA stability	+	[29]
NGF	3’UTR	AU-rich element	mRNA stability	+	[29]
Neuritin 1	3’UTR	AU-rich element	Localization	+	[9]
Neuritin 1	3’UTR	AU-rich element	Localization	+	[14]
Neuritin 1	3’UTR	AU-rich element	mRNA stability	+	[36]
NF–1	Intron	AU-rich element	Alternative splicing	+	[37]
NF–1	Intron	AU-rich element	Local transcription elongation	+	[38]
Neuroserpin	3’UTR	AU-rich element	mRNA stability	+	[39]
NT-3	3’UTR	AU-rich element	mRNA stability	+	[29]
NOVA–1	3’UTR	AU-rich element	mRNA stability and Translation	+	[40]
MSI1	3’UTR	AU-rich element	mRNA stability	+	[41]
Kv1.1	coding region	U-rich element	Translation	+	[42]
SATB1	3’UTR	AU-rich element	mRNA stability	+	[43]
SOD1	3’UTR	AU-rich element	mRNA stability	+	[44]
Tau	3’UTR	U-rich element	Transport	+	[45]

## Data Availability

Not applicable.
